# Research on the sentiment recognition and application of allusive words based on text semantic enhancement

**DOI:** 10.1371/journal.pone.0308944

**Published:** 2024-11-04

**Authors:** Xiaomin Li, Hao Wang, Bin Shi, Wenru Bu

**Affiliations:** 1 School of Information Management, Nanjing University, Nanjing, China; 2 Jiangsu International Joint Informatics Laboratory, Nanjing University, Nanjing, China; Universidad de Oviedo, SPAIN

## Abstract

In the era of digital intelligence empowerment, the data-driven approach to the mining and organization of humanistic knowledge has ushered in new development opportunities. However, current research on allusions, an important type of humanities data, mainly focuses on the adoption of a traditional paradigm of humanities research. Conversely, little attention is paid to the application of auto-computing techniques to allusive resources. In light of this research gap, this work proposes a model of allusive word sentiment recognition and application based on text semantic enhancement. First, explanatory texts of 36,080 allusive words are introduced for text semantic enhancement. Subsequently, the performances of different deep learning-based approaches are compared, including three baselines and two optimized models. The best model, ERNIE-RCNN, which exhibits a 6.35% improvement in accuracy, is chosen for the sentiment prediction of allusive words based on text semantic enhancement. Next, according to the binary relationships between allusive words and their source text, explanatory text, and sentiments, the overall and time-based distribution regularities of allusive word sentiments are explored. In addition, the sentiments of the source text are inferred according to the allusive word sentiments. Finally, the LDA model is utilized for the topic extraction of allusive words, and the sentiments and topics are fused to construct an allusive word-sentiment theme relationship database, which provides two modes for the semantic association and organization of allusive resources. The empirical results show that the proposed model can achieve the discovery and association of allusion-related humanities knowledge.

## 1. Introduction

The digital humanities (DH) is a new interdisciplinary research paradigm in which computational technologies and methods are applied to study various cultural phenomena in humanities discipline [1–3]. Lately, there has been an increasing research interest in the exploitation of various computational data analysis methods for the organization and association of humanities resources, including cultural heritage [4–7], geographical information [8–10], and literature art [11,12]. In these studies, it is shown that digital technologies have provide new methods, new means and new perspectives for humanities scholars to make humanities resources digitally available, analyzable and organizable.

Allusion, a representative humanities resource, serves as a witness to the long history of the Chinese nation and a tangible embodiment of the nation’s cultural memory and transmission [13]. Since the 18th National Congress of the Communist Party of China, the Chinese President *Xi Jinping* has frequently drawn upon authoritative works in a series of important speeches [14], articles [15], and conversations [16] with the public to articulate his thoughts, stances, and perspectives. This act has fostered the creative transformation and innovative development of allusion culture, thereby strengthening the Chinese populace’s attention for it [17,18]. In this vein, driven by the digital humanities (DH), allusion entity recognition has attracted considerable attention to identify and locate allusion entities in sentences of Chinese ancient poetry [13,19]. In another line of research, the allusion visualization applying Virtual Reality, Augmented Reality, and knowledge graph technologies was studied [20–22]. Moreover, allusion citation behavior analysis from the perspective of informatics was explored [23]. As a result, there is a growing body of research investing and exploring the computational methods and technologies of the digital humanities to analyze and organize allusive resource. However, existing research has been done either on allusions in the Chinese ancient poetry [24–26] or external attribute display of allusions through visualization tools [22,27]. To the best of our knowledge, the semantic attributes and associations of allusions have not been thoroughly investigated yet, resulting in the public’s familiarity with some common allusions such as “*精忠报国*” (be loyal and serve the country), “*草木皆兵*” (every bush and tree looks like an enemy described as a state of extreme nervousness), and “*鸿门宴*” (the *Hongmen* Banquet symbolizing a treacherous and dangerous situation). Conversely, lesser-known allusions such as “*工力悉敌*” (force and skill do match), “*箪食壶浆*” (welcome soldiers with food and drink), and “*稻粱谋*” (seek food and clothing) remain obscure.

In this research, we conduct a comprehensive study to organize and association allusions by applying computational semantic methods. We extracted two semantic features: sentiments and topics. More specifically, our main research hypotheses are as follows:

H1: The text semantic enhancement of allusion data can effectively improve the accuracy of the sentiment recognition of allusive words.

H2: Allusive word sentiments follow a distribution of more positive words than negative words.

H3: In certain circumstances, the emotion of allusive words can be utilized to infer the emotion of the text.

H4: The inclusion of the themes of allusive words enables the effective organization of allusive resources at the semantic level.

To verify these research hypotheses, this study proposes a framework upon which to build a model of the sentiment recognition and application of allusive words. Allusive words are taken as the research object primarily to achieve their automatic sentiment recognition, as well as to discover the sentiment regularity of, and the semantic associations between, allusive words based on their theme and sentiment. First, the performances of different deep learning algorithms are compared, and the best is chosen for allusive word sentiment recognition and prediction based on text semantic enhancement. Thereafter, the statistical analysis of the sentiment distribution regularity of allusive words is performed. Finally, the themes of allusive words are extracted by the LDA model, and the semantic associations between allusive words and their sentiments, as constructed by an allusive word-theme and sentiment relationship database, are displayed via Neo4j.

In summary, the main contributions of this research are as follows:

1. A deep learning algorithm is used for the automatic recognition of allusive word sentiments in allusive resources.
2. Based on sentiment recognition, statistical analysis is conducted to discover some regular humanities knowledge.
3. The incorporation of the thematic elements of allusive words is found to facilitate the association and organization of allusive resources.

The remainder of this article is organized as follows. Section 2 summarizes previous studies related to allusive resources and sentiment recognition. Section 3 describes the proposed model and methodology used in this research. Sections 4 and 5 present the empirical results and the discussion of the analysis. Finally, the conclusions, implications, and recommendations for future studies are presented in Sections 6 and 7.

## 2. Related work

The literature related to this study is reviewed regarding the two aspects of (1) allusive resources and (2) sentiment recognition.

### 2.1 Allusive resources

The use of allusion is a common phenomenon in Chinese literary works, in which the ancients, ancient stories, or old sayings are invoked to euphemistically present the author’s inner world. From ancient times to the present, allusions have been created and repeatedly quoted, thus forming a unique allusion culture in China. Allusive resources contain four key elements, namely the allusive source, allusive word, allusive meaning, and allusive example, and the current research on allusion mainly focuses on these four aspects. Ren et al. [28] focused on the allusions contained in the ancient Chinese classic *Meng Qiu*, and took the allusion which Sun Kang read absorbedly with the reflected light of snow and *Chen Yin* studied hard by the light of bagged fireflies as an example to examined a series of influences on Japanese literature after the introduction of *Meng Qiu* to Japan. van Ess [29] analyzed the literary works and allusions in Three Biographies of the *Hou Hanshu*, and help readers understand the cases hidden allusions. Wu and Fitzgerald [30] explored three discursive techniques of indirection used by Chinese social media users to express political criticism in the context of censorship, and found that users challenge authorities’ official narratives of events via the creative use of quotation, allusion, and irony. Chung [31] designed an explanatory framework of three cultural drivers including allusion and six tactics for the analysis of China’s psychological warfare. Nikolaeva [32] proved that linguistic creativity is realized in English texts via various forms of manifestation of Chinese national communicative identity, including proverbs, allegories, analogies, hints, and allusions rooted in Chinese history, philosophy, and folk experience. Lin [33] investigated the discourse of a long-lasting social debate over the safety of genetically modified food in China, and found that the two opposing sides use allusions to express their opinions.

In summary, while progress has been made in terms of the investigation of allusive resources, allusive resources have mostly been manually analyzed via qualitative methods, and the conclusions and regularities are subjective and biased. Moreover, previous research has been limited in depth and breadth. The design of an efficient model with which to automatically and quantitatively analyze allusive resources is also needed.

### 2.2 Sentiment recognition

Sentiment recognition, which is currently a popular research topic, is applied to a wide variety of data types, such as texts, images, sounds, etc., in different domains.

According to the authors’ observation, the task of sentiment recognition remains mostly text-specific, and involves different domains including social media, medicine, government, citation, games, etc. Dong et al. [34] applied the deep learning DC-BiGRU-CNN model to the irony recognition of Chinese social comments, which overcame the difficulty encountered by existing machine learning algorithms in discriminating ironic tones. Zhou et al. [35] proposed the ensemble correction (EC) model to extract features from different word embedding models and reduce computational complexity for public opinion analysis. Aiming at the emotion recognition of Chinese medical reviews, Jin et al. [36] proposed a hybrid deep neural network model called TBLC-rAttention. Zhang et al. [37] constructed a public emergency-oriented emotion lexicon for the emotion evolution of public events, and explored the mechanism by which the government’s information release strategy influences the contagion evolution of negative emotions. Wang et al. [38] explored the linguistic patterns of emotional expression in the citation context, based on which they recognized the sentimental polarity of the citation context. Yan et al. [39] used a large data set of PubMed Central (PMC) full-text publications, and analyzed the citation sentiment in more than 32 million citances within PMC; they revealed citation sentiment patterns at both the journal and discipline levels. Aljuaid et al. [40] classified citations into binary classes, and used metadata, content, citation counts, cue words or phrases, sentiment analysis, keywords, and machine learning approaches for citation classification. Dehdarirad and Yaghtin [41] investigated whether female and male authors in the life sciences and biomedicine fields differed in their citation and citation sentiment tendencies via a combination of homophily analysis, regression analysis, and chi-square tests. Öhman and Kajava [42] introduced a publicly available gamified web-based annotation platform for 51 unique sentiments and emotion annotation at the sentence level.

In addition to text, images and videos are also used for sentiment classification. Zhang and Xu [43] proposed an end-to-end deep neural network for image emotion recognition that leverages emotion intensity learning, and evaluated the emotion recognition and sentiment classification abilities of the proposed network on different benchmark datasets. Yang et al. [44] leveraged emotional concepts as an intermediary to create a bridge between images and emotions for image emotion recognition. Li et al. [45] transformed raw physiological signals in each channel into a spectrogram image to capture time and frequency information, and applied attention-based long short-term memory recurrent neural network (LSTM-RNN) models to learn the best temporal features for emotion prediction. Chen et al. [46] collected and annotated a new multi-language multimodal video emotion database named HEU Emotion. This database was recorded in a multimodal synchronous way and can be directly used for multimodal emotion recognition experiments. Russo et al. [47] proposed a new cochleogram-based system for the detection of affective musical content, in which a music audio signal is processed by a detailed biophysical cochlear model to obtain an output that closely matches the characteristics of human hearing. Abbaschian et al. [48] investigated the field of discrete speech emotion recognition via a multi-aspect comparison between practical neural network approaches. Chatterjee et al. [49] introduced a comprehensive approach for human speech-based emotion analysis in which a one-dimensional (1-D) convolutional neural network (CNN) was implemented to learn and classify the emotions associated with human speech.

The sentiment classification of single-modal data has been investigated by numerous researchers, some of whom have also explored cross-modal sentiment analysis. Moreover, multimodal sentiment recognition has proven to be feasible. Kumar et al. [50] proffered a hybrid deep learning model for the prediction of fine-grained sentiments in real-time multimodal data; the model includes four modules, namely discretization, text analytics, image analytics, and decision modules. Han et al. [51] proposed a novel cross-modal emotion embedding framework called EmoBed, which can leverage the knowledge from other auxiliary modalities to improve the performance of an emotion recognition system at hand. Tuncer et al. [52] presented a multi-level handcrafted feature generation-based automated emotion classification model using electroencephalogram (EEG) signals, and validated the developed model using three databases. These studies have provided a variety of useful models and algorithms for the solution to sentiment recognition. Table 1 shows some of the key literatures.

**Table 1:** Some key literatures of review helpfulness on allusive resources and sentiment classification.

Author	Proposed method	Research object (allusive words)	Research method (Deep learning)	Research objective (Organization and association)
[33]	investigates the discourse of a long-lasting social debate over the safety of genetically modified food in China, and found that the two opposing sides used allusions to express their opinions	Π	Ο	Ο
[32]	proves that linguistic creativity was realized in English texts through various forms of manifestation of Chinese national communicative identity: proverbs, allegories, analogies, hints, and allusions rooted in Chinese history, philosophy, and folk experience	Π	Ο	Ο
[35]	proposes the ensemble correction (EC) model to extract features from different word embedding models and reduce computational complexity for public opinion analysis	Ο	Π	Ο
[41]	investigates whether female and male authors in the life sciences and biomedicine fields differed in their tendencies of citation and citation sentiment via a combination of homophily analysis, regression analysis, and a chi-square test	Ο	Π	Ο
[49]	introduces a comprehensive approach for human speech-based emotion analysis, in which the 1-D CNN was implemented to learn and classify the emotions associated with human speech	Ο	Π	Ο
Our study	proposes a model of allusive word sentiment recognition and application based on text semantic enhancement	Π	Π	Π

As is evident from the preceding literature review, the current research on allusive resources mainly focuses on employing traditional humanities research paradigms, thus leaving unexploited the application of automation technology and qualitative analysis methods to allusive resources. Hence, this study proposes a model of the emotion recognition and application of allusive words based on text semantic enhancement. Emotion recognition technology is applied to allusive resources to determine the emotional polarity of allusive words, and the relationships between the elements in allusive resources are leveraged to realize knowledge mining and semantic organization.

## 3. Methodology

### 3.1 Task definition

We analyze allusive resources from two aspects, i.e., the sentiment recognition of allusive words and application research around sentiment of allusive words. The task of sentiment recognition of allusive words can be formalized as a binary classification task. This approach is justified for two reasons. First, allusive words are typically cited to express opinions and positions, thereby enhancing persuasive power [53], whereas neutral sentiments generally describe objective facts. Second, the dataset used in this study has been partially labeled with allusive word sentiments by domain researchers, and the number of neutral sentiments is significantly smaller compared to positive and negative ones with a ratio close to 30:1. Therefore, the sentiments of allusive words are categorized as positive and negative. The input for the task is a text corpus or corpus combination on allusive words, and the output is one of 2 classes that represent the sentiment of allusive words, namely *Positive* and *Negative*. Moreover, the effects of different deep-learning methods for sentiment classification also are compared.

For the task of application research around sentiment of allusive words, we analyzed this task from two aspects, i.e., the regularity discovery and semantic organization and association. For the regularity discovery, the overall and time-transformation distributions of allusive word sentiment are explored. In addition, the text sentiment inference is conducted according to the allusive word sentiment. For the semantic organization and association, the LDA model is leveraged to extract the topics of allusive words, and fused with sentiment of allusive words to construct topic emotion relationship database for sematic organization and association of allusive resources.

### 3.2 Model overview

**Fig 1:**
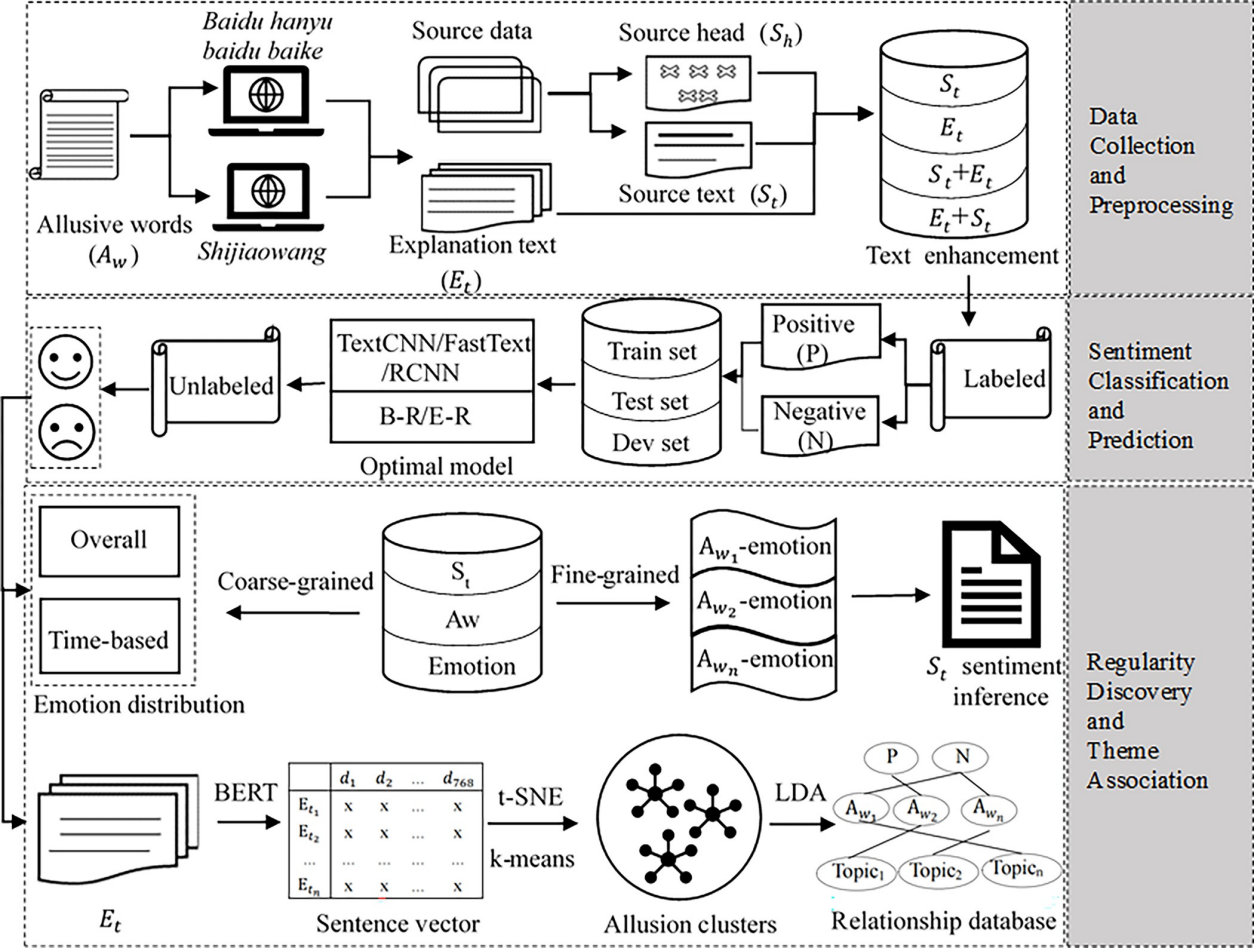
The overview of the model framework.

As shown in Fig 1, the proposed model framework includes the following three main procedures.

- Data collection and preprocessing. At this stage, allusive words (*A*_*w*_) and source contents, including the source head (*S*_*h*_) and source text (*S*_*t*_), are first crawled from Shijiaowang. The explanation text (*E*_*t*_) of allusive words is also crawled from Baidubaike and Baiduhanyu for text semantic enhancement. Then, the different text data are combined and four types of corpora are generated, namely *S*_*t*_, *E*_*t*_, *S*_*t*_+*E*_*t*_, and *E*_*t*_+*S*_*t*_.
- Sentiment classification model selection and prediction. The four corpora are first divided into training data, test data, and dev data, respectively. The performances of multiple deep learning algorithms for the sentiment recognition of allusive words are then compared, and the results are analyzed to verify the correctness of Hypothesis 1 and make sentiment predictions.
- Regularity discovery and theme analysis. Relying on the dichotomous relationships between allusive words and their source text, explanatory text, and sentiments, the overall and time-based distributions of allusive word sentiments are first analyzed at the coarse-grained level to verify Hypothesis 2. Next, certain circumstances that could infer text sentiments are explored at the fine-grained level according to the sentiments of allusive words to verify Hypothesis 3. In addition, sentence vectors (*V*_*s*_) of explanatory text are generated through Bidirectional Encoder Representations from Transformer (BERT), and the dimension is reduced by t-SNE. Then, the k-means algorithm and latent Dirichlet allocation (LDA) are utilized for clustering and topic extraction to determine the theme to which the allusive word belongs, and a sentiment-topic relationship database is then constructed for the semantic organization of allusive resources.

### 3.3 Data collection and preprocessing

The *Shijiaowang* (http://www.zhscwx.com/diangu/) website was used as the data collection platform. It is a knowledge integration website dedicated to the transmission and popularization of humanities knowledge, including Chinese poetry, allusions, and couplets, and provides comprehensive and accurate humanities data for relevant researchers. We developed a Python program to crawl data from the *Diangudaquan* subpage of *Shijiaowang*, and collected publicly available data about allusive resources including allusive words (*A*_*W*_) and their source heads (*S*_*h*_), source texts (*S*_*t*_), and emotion labels.

After data cleaning operations such as data checking and missing value elimination, 36,080 allusive words (*A*_*W*_) and their corresponding source head (*S*_*h*_), source text (*S*_*t*_), and emotion label were obtained.

Via the further observation of the data, it was found that not every allusive word had a sentiment label. Hence, the allusive words were divided into labeled and unlabeled data. Statistically, there were 9,597 labeled allusive words, among which 5,073 were positive and 4,254 were negative, and 26,483 unlabeled allusive words. These data were utilized for sentiment recognition model training and sentiment prediction.

### 3.4 Text semantic enhancement

The source text (*S*_*t*_) of allusive words originates from different historical periods. There are various genres, including chronicles, poetry, novels, essays, etc., and the different genres directly result in inconsistent grammatical structures and sentence forms. Chronicles and poems mainly focus on single words with concise lines and special uses, such as inversion and omission, while novels and essays mainly focus on words that are easy to understand and rich in semantic information. If the *S*_*t*_ is used solely for allusive word sentiment recognition, the recognition may not be as effective as it should be due to the variation of syntaxes and sentence patterns. Moreover, the *S*_*t*_ is generally short in length, and semantic information is missing. Therefore, in this study, the explanation text (*E*_*t*_) was introduced for text semantic enhancement to improve the effect of sentiment recognition. The *E*_*t*_, as the name implies, is leveraged to explain the deeper meaning of the allusive words with a uniform syntax and rich contextual semantic information. Similarly, we also developed Python programs to crawl the *E*_*t*_ corresponding to 36,080 allusive words from *Baidu baike* (https://baike.baidu.com/) and *Baidu hanyu* (https://hanyu.baidu.com/). According to our observation, there are cases where the *E*_*t*_ of an allusive word does not exist in *Baidu baike*, but exists in *Baidu hanyu*, or vice versa, so that data were collected from two websites to ensure integrity. And we state that all data collection and analyses undertaken in this study complied with the data use terms and conditions of *Shijiaowang*, *Baidu baike* and *Baidu hanyu*. An example of the data is provided in Table 2.

**Table 2:** An example of the data.

*A*_*w*_	*S*_*h*_	*S*_*t*_	*E*_*t*_ (for text enhancement)	Emotion
*温故知新* (gain new insights through reviewing old material)	*《论语·为政》* (the Article recording the Confucius’ principles of politics)	*温故而知新*, *可以为师矣。* (One who likes to review history and may acquire new knowledge, and so it is possible to turn into a Master.)	*指温习已学过的知识*, *获得新的理解和体会。也指吸取历史经验*, *认识现在。也指重温历史*, *可以认识现在。描写学习方法。* (Someone could gain new understanding and experience by reviewing old knowledge. It also refers to revisiting history so that you can know the present. It is usually used to describe the method of learning.)	Positive

### 3.5 Emotion recognition based on deep learning algorithms

Emotion is an internal subjective experience and a special form of the real world formulated by human beings. Emotion recognition aims to determine the emotions embedded in objective things, which can be converted to an emotion classification task. Minaee et al. [54] categorized text classification models based on neural network architectures, including feed-forward neural networks, CNN-based models, RNN-based models, and so on. Therefore, in this study, the variants of the top three models (i.e., FastText, TextCNN, and Recurrent Convolutional Neural Network (RCNN)) were chosen as baselines, and the best baseline model was combined with BERT and Enhanced Representation through kNowledge IntEgration (ERNIE) to train the sentiment classification model for allusive words.

#### 3.5.1 Baselines

*TextCNN*. TextCNN [55] is a feed-forward neural network consisting of an input layer, convolutional layer, pooling layer, and fully connected layer. TextCNN performs text classification by first inputting a text sequence into the model and obtaining the word vector of each word through the input layer. The convolution layer then extracts the text features, and the pooling layer uses max-pooling to ensure that a fixed-length text feature vector is obtained. The pooled feature vector enters the fully-connected layer and accesses a softmax layer to output the probability of each category.

*FastText*. FastText [56] is a fast text classification algorithm that does not require a word vector to be trained in advance; it can train the word vector itself. The model is characterized by a fast training and testing speed while maintaining high accuracy. After a piece of text is input into the FastText model, the vector mean of all words is obtained and passed into the hidden layer to perform nonlinear transformation. It finally reaches the output layer using the softmax function to map the probabilities of the word belonging to different categories.

*RCNN*. The RCNN for text classification [57] is similar to TextCNN, but the main difference lies in the convolutional layer. The convolutional layer of TextCNN is a CNN, whereas that of RCNN is replaced with a bidirectional RNN. Similarly, the model consists of an input layer, convolutional layer, pooling layer, and fully connected layer. First, a text sequence is input, and the left and right sides use a bidirectional RNN to respectively learn the left and right context representations of the current word, which are connected with the vector of the current word itself as the input of the convolutional layer. After the convolutional layer, the spliced vector is nonlinearly mapped to a lower dimension, and the semantic representation of all words in the text is obtained. Then, a max-pooling layer is used so that the value of each position in the vector takes the maximum value on all time series to obtain the final feature vector. Finally, the softmax layer is accessed to obtain the probability of each category. In this study, bidirectional long short-term memory (BiLSTM) was selected for use in the bidirectional RNN text classification experiments.

#### 3.5.2 Optimization models

*BERT-based optimized model*. BERT [58] is a language model built based on the bidirectional transformer proposed by Google in 2018, and is obtained by training the masked language model (LM) and next sentence prediction (NSP). The masked LM mechanism aims to randomly mask some characters in the corpus, and then to train the model to predict the masked characters. The actual process is divided into three steps. First, 15% of the words are randomly selected from each sentence. Then, the selected words are replaced with [Mask] with 80% probability, they are replaced with any word with 10% probability, and they are not replaced with 10% probability. Finally, the corpus is trained after masking. The role of NSP is to understand the relationship between sentences and determine whether sentence A is the next statement of sentence B. In addition, NSP adds the character [CLS] at the beginning of each paragraph to store classification-related information, and adds the character [SEP] between sentences to divide the sentences. Google provides a pre-trained BERT model. In this study, the 768-dimension pre-trained BERT model was used to obtain word vectors, which were utilized as the input layer of the classification model for text classification.

**Fig 2:**
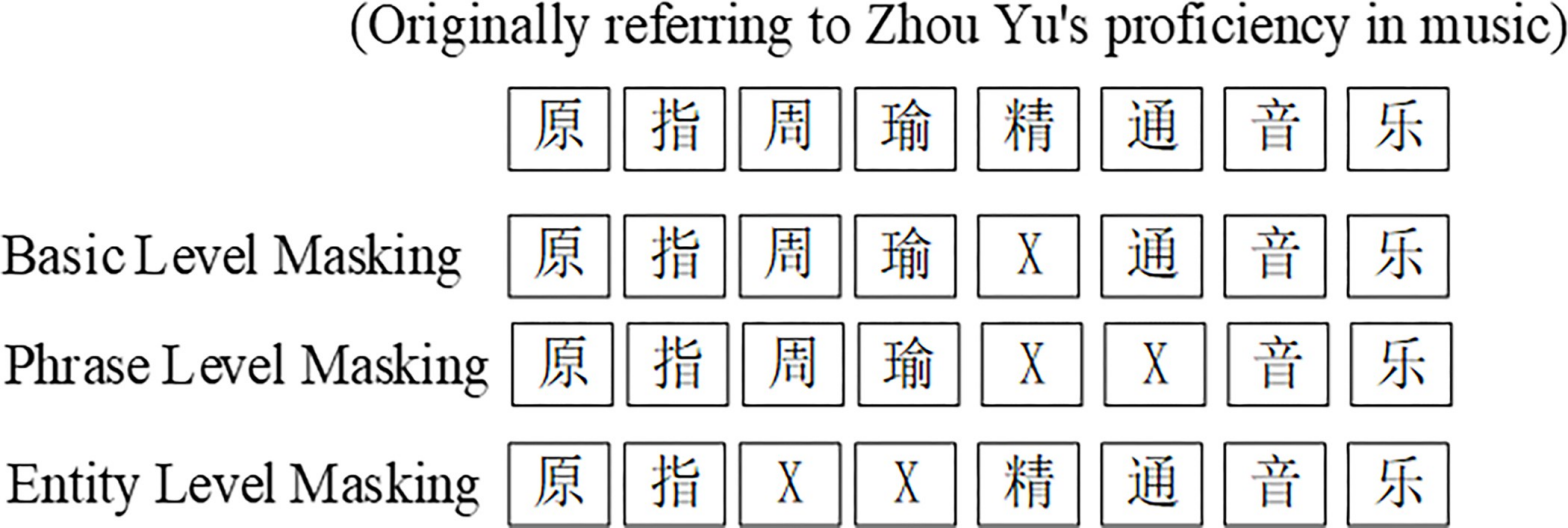
The ERNIE masking strategy.

*ERNIE-based optimization model*. ERNIE [59], which is similar to the BERT framework, differs mainly in the pre-training task. The first difference is related to the pre-training data; in addition to the Wikipedia corpus used by BERT, ERNIE also includes the addition of multi-source corpora such as Baidubaike, Baiduhanyu, and Baidutieba. The expansion of the pre-training corpus enhances the semantic representation of the model. The second difference is the masking strategy; ERNIE adopts a random masking strategy, and the masking principle is presented in Fig 2.

**Fig 3:**
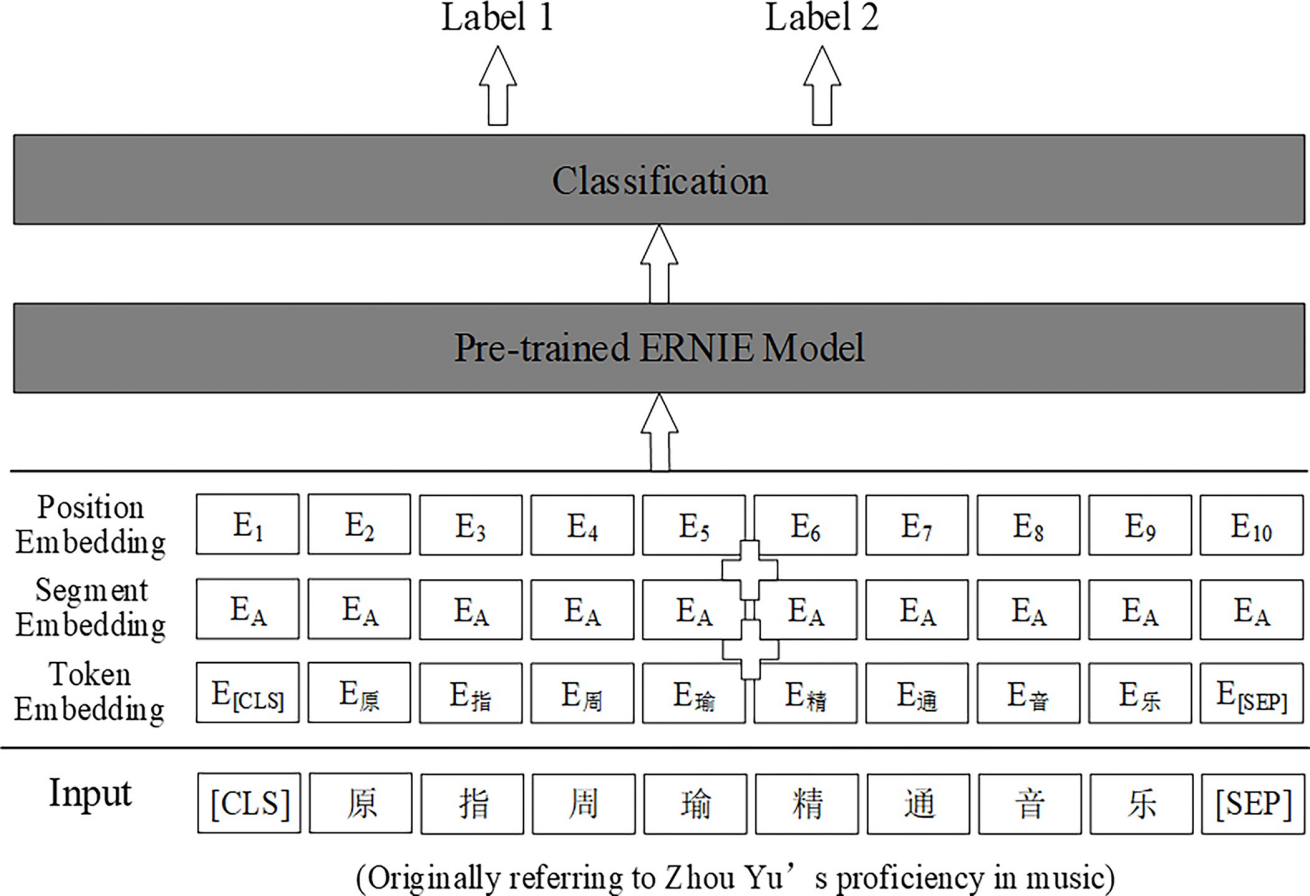
The diagram of the ERNIE model.

Unlike BERT, which only masks words via basic-level masking, ERNIE adds phrase and entity masks called phrase-level masking and entity-level masking, respectively. It builds a multi-level masking strategy to be more suitable for the Chinese corpus. Moreover, the model can implicitly learn the a priori semantic knowledge of masked phrases and entities to enhance the generalization ability of the model and improve the recognition effect of downstream tasks. In the ERNIE model, a large amount of the general corpus is trained to generate a pre-training model. Similarly, the word vector of the text data is obtained using the pre-trained ERNIE model, and is used as the input layer of the subsequent classification model to achieve text classification. Fig 3 displays the principle of ERNIE for text classification.

### 3.6 Cluster algorithms and topic analysis methods

#### 3.6.1 Cluster algorithms

*t-SNE*. t-distributed stochastic neighbor embedding (t-SNE) [60], a nonlinear dimensionality reduction algorithm, is suitable for reducing high-dimensional data to two or three dimensions. The fundamental idea is that if two vectors are similar in a high-dimensional space, they should also be close to each other in the low-dimensional space. t-SNE excels at preserving the relative spatial position of the original data when downscaling. In our work, t-SNE was employed to reduce the high dimensional semantic vectors of allusion words generated by BERT to two-dimensional embedding vectors and visualize them, thereby facilitated the following clustering.

*k-Means*. *k*-means [61] is a typical division-based unsupervised clustering algorithm known for its efficiency and widespread used in big data processing. In this study, *k*-means was leveraged to cluster allusion words. Moreover, *k*-means was improved to enhance its performance. Specifically, the corpus of *E*_*t*_ was encoded to 768-dimensional semantic vector by applying BERT, and further reduced to 2 dimensions by t-SNE. This improvement replaced the original one-hot vector representation of text in k-means clustering.

To determine the optimal number of clusters *k*, the elbow method was selected. This method calculates SSE (the Sum of Square Errors), which represents the sum of distances from all sample points in the dataset to their cluster centers. The optimal *k* value corresponding to the significant slowdown of the decline of SSE was chosen. Finally, the optimal number of clusters *k* was determined to be 30.

#### 3.6.2 Topic analysis methods

To explore the semantic features of allusion words, LDA [62] was employed to extract topics of allusion words over the corpus of *E*_*t*_ in our work. LDA is a document-topic generation model consisting of three hierarchical structures: documents, topics, and words. The basic idea is that a document can contain multiple topics, and each word in the document is generated from one of the topics. The document-to-topic distribution is polynomial, as is the topic-to-word distribution.

The *Gensim* package in Python was used to extract topics. To determine the optimal number of topics *k*, the perplexity and topic distance index were chosen as evaluation metrics. Models of 1–15 topics were built to calculate the perplexity. And the lower the perplexity, the better the topic extraction effect. The number of topics with the lowest perplexity was 13. Moreover, pyLDAvis, a python library for interactive topic model visualization, was utilized to interpret topic results by visualizing distances among topics in our study.

## 4. Empirical research

In this section, an experiment on the emotion recognition of allusive words was conducted. First, the performances of multiple classification algorithms are compared. Then, the best algorithm is chosen for the verification of Hypothesis 1, as well as sentiment prediction.

### 4.1 Sentiment recognition model training

With the aim of verifying Hypothesis 1, the *E*_*t*_ was first introduced on the basis of the St of allusive words for text semantic enhancement. Specifically, four corpus combinations were constructed, including *S*_*t*_, *S*_*t*_+*E*_*t*_, *E*_*t*_, and *E*_*t*_+*S*_*t*_. Then, the 9,597 pieces of labeled data were divided into training, testing, and development sets according to the ratio of 6:2:1. The remaining 26,483 pieces of unlabeled data were utilized for prediction. Finally, the sentiment recognition model training performances of three baseline models, including TextCNN, FastText, and RCNN, were compared, and the best model was selected for optimization. Further, the optimal model and optimal corpora are determined according to the performance of the optimized algorithms. The experiments were all conducted on a server with two RTX 2,080 GPUs. The programming language was Python 3.7, and the deep learning framework was PyTorch 1.1.

#### 4.1.1 Baselines

Three baselines, including TextCNN, FastText, and RCNN, were employed to perform the sentiment classification experiment, and their performances were compared. Thereafter, the model with the best performance was chosen for optimization. The learning rate was set to 0.001, adam was employed as the optimizer, and the batch size was set to 128. Moreover, the number of training epochs was 100. Additionally, the early stopping technique was used; training was stopped when the loss value did not decline for 10 consecutive rounds. The results are presented in Table 3.

**Table 3:** The baseline classification results.

Corpus	Baselines	ACC	Macro_P	Macro_R	Macro_F1	Positive			Negative		
						P	R	F1	P	R	F1
*S*_*t*_	TextCNN	68.13	67.92	67.89	67.90	70.17	70.99	70.58	65.68	64.79	65.23
	FastText	66.09	65.90	65.52	65.55	66.99	73.02	69.88	64.82	58.01	61.23
	RCNN	69.64	69.46	69.47	69.47	71.91	71.57	71.74	67.00	67.38	67.19
*S*_*t*_+*E*_*t*_	TextCNN	72.71	73.61	73.14	72.64	79.66	64.48	71.27	67.57	81.80	74.01
	FastText	70.63	70.55	70.49	70.51	71.51	73.21	72.35	69.59	67.76	68.67
	RCNN	74.84	74.80	74.86	74.81	76.81	74.60	75.69	72.79	75.11	73.93
*E*_*t*_	TextCNN	86.04	86.04	85.73	85.86	86.03	88.97	87.48	86.06	82.49	84.24
	FastText	87.03	86.95	87.28	86.99	91.01	84.70	87.74	82.89	89.86	86.24
	RCNN	87.40	87.33	87.66	87.36	91.50	84.89	88.07	83.16	90.44	86.64
*E*_*t*_+*S*_*t*_	TextCNN	85.10	85.44	85.52	85.10	91.26	79.94	85.23	79.63	91.10	84.98
	FastText	86.82	87.04	86.49	86.66	85.51	90.89	88.12	88.58	82.09	85.21
	**RCNN**	**87.50**	**87.44**	**87.41**	**87.42**	**88.15**	**88.66**	**88.41**	**86.73**	**86.15**	**86.44**

The RCNN model was found to outperform all the baseline models. Also, in terms of corpora combination, the recognition effect of the RCNN, from highest to lowest, was found to be as follows: *E*_*t*_+*S*_*t*_>*E*_*t*_>*S*_*t*_+*E*_*t*_>*S*_*t*_. In contrast, for TextCNN and FastText, the recognition effect, from highest to lowest, was as follows: *E*_*t*_>*S*_*t*_+*E*_*t*_>*E*_*t*_+*S*_*t*_>*S*_*t*_. To further verify the optimal corpus combination, the strongest-performing baseline, RCNN, was selected. Instead of using randomly initialized word embedding to perform model optimization, the model input layer was respectively replaced with the pre-trained BERT and ERNIE contextualized language models. Then, the effects of the optimized models were compared to verify whether Hypothesis 1 holds. Because the recognition effects of St and *S*_*t*_+*E*_*t*_ were much lower than those of *E*_*t*_ and *E*_*t*_+*S*_*t*_, focus was placed on comparing the recognition effects of the *E*_*t*_ and *E*_*t*_+*S*_*t*_ corpora in the subsequently optimized models.

#### 4.1.2 Optimization models

The RCNN was selected and respectively combined with BERT and ERNIE to obtain the B-R (BERT-RCNN) and E-R (ERNIE-RCNN) models. Specifically, the pre-trained BERT and ERNIE models were respectively used to obtain the word embeddings as the input layers, which were input into the RCNN model for the sentiment recognition of allusive words. The results are presented in Table 4.

**Table 4:** The classification results of the optimized models.

Corpus	Model	ACC	Macro_P	Macro_R	Macro_F1	Positive			Negative		
						P	R	F1	P	R	F1
*E*_*t*_	B-R	91.93	92.44	91.44	91.77	89.59	96.48	92.91	95.30	86.41	90.63
	**E-R**	**93.07**	**92.95**	**93.27**	**93.04**	**95.90**	**91.25**	**93.52**	**89.99**	**95.28**	**92.56**
*E*_*t*_+*S*_*t*_	B-R	92.86	92.78	92.93	92.84	94.53	92.05	93.27	91.04	93.81	92.40
	**E-R**	**93.85**	**93.90**	**93.74**	**93.81**	**93.44**	**95.25**	**94.34**	**94.35**	**92.23**	**93.28**

It was found that the B-R model improved the accuracy of the baseline RNN model by 4.53% and 5.36% for the *E*_*t*_ and *E*_*t*_+*S*_*t*_ corpora, respectively, while the E-R model contributed to accuracy improvements of 5.67% and 6.35% for the *E*_*t*_ and *E*_*t*_+*S*_*t*_ corpora, respectively. Such an obvious performance increase reflects the usefulness of model optimization. This also provides clear evidence that the E-R model achieved the strongest performance for the sentiment classification of allusive words. Our explanation for this is that the dataset in this study consists of combinations of *E*_*t*_ and *S*_*t*_, which involve two different grammatical and syntactic styles. The ERNIE pre-training model effectively learns lexical structure, syntactic structure, and semantic information of *E*_*t*_+*S*_*t*_, generating the hierarchical semantic vectors that are then passed to the RCNN. The RCNN combines the advantages of CNN and RNN, allowing it to capture both local and global features for optimal classification effects. Additionally, the E-R model has strong robustness and generalization abilities, achieving satisfactory recognition results even with the limited labeled data available in this study.

As can be seen from the results reported in Table 3, the accuracy for positive allusive words and the recall value for negative allusive words of the E-R model on the *E*_*t*_ corpora were abnormally high, so the results of recognition were unbalanced. In contrast, the E-R model on the *E*_*t*_+*S*_*t*_ corpora was obviously balanced, and the overall recognition effect was the best. Therefore, the corpora recognition performance, from highest to lowest, was *E*_*t*_+*S*_*t*_>*E*_*t*_>*S*_*t*_+*E*_*t*_>*S*_*t*_, which indicates the correctness of Hypothesis 1.

Taking into account the specific content of the corpora, the reason for achieving the best sentiment classification performance was also explored through text semantic enhancement. The *S*_*t*_ corpora is condensed and brief, encompassing various genres, such as literary texts, poetries, and novels. In contrast, the *E*_*t*_ corpus is more uniform in terms of the syntactic type and contains more richer contextual information. By introducing *E*_*t*_, the sentiment recognition model can better understand and capture the sentimental features in sentences, thereby enhancing its semantic comprehension and contextual awareness abilities.

The experimental results demonstrate that the corpora combination with predominantly *E*_*t*_ and supplemented by *S*_*t*_ was optimal. Therefore, for the unlabeled allusive words, the trained E-R model was applied to the *E*_*t*_+*S*_*t*_ corpora for the sentiment prediction of allusive words.

### 4.2 Sentiment prediction

Further, the 26,483 unlabeled data were used as the prediction corpora and were stitched into semantically enhanced text in the order of *E*_*t*_+*S*_*t*_. The trained E-R model was utilized for the prediction of allusive word sentiments. To evaluate the model’s zero-shot learning performance, 500 allusive words were randomly selected for evaluation. Among these, 28 allusive words were misrecognized: 9 negative allusive words were misidentified as positive, and 19 positive allusive words were misidentified as negative. The zero-shot learning performance reached 94.4%, indicating that the sentiment prediction result is credible.

## 5. Emotion distribution and theme association

The allusive resources were mined and organized through the dichotomous relationships that exist between the allusive words and the *S*_*t*_ corpora, the *E*_*t*_ corpora, and sentiments for knowledge discovery and association. Regarding the allusive word emotion distribution, at a coarse-grained level, the overall distribution of allusive word emotions was first explored using the binary relationship between allusive words and emotions. The binary relationship between allusive words and the *S*_*t*_ corpus was then incorporated to investigate the distribution of allusive word emotions based on time transformation. Finally, some regular knowledge was gleaned via statistical analysis. At a fine-grained level, the text sentiment was inferred via the dichotomous relationship between allusive word-*S*_*t*_ corpus and allusive word-sentiment. Regarding theme association, allusive words were first clustered to form cluster groups based on the binary relationship between allusive words and the *E*_*t*_ corpus. Next, the themes of cluster groups were extracted for the semantic organization and association of allusive resources by fusing the binary relationships between allusive words and sentiments.

### 5.1 Emotion distribution and emotion inference

This subsection discusses the distribution regularity of emotion, as well as knowledge inference. This includes the comparison and analysis of allusive words based on the number and ratio of emotions, namely the overall distribution of emotions in allusive words and their distribution over time, as well as textual emotion inference. The subsection supplements the status that the prior research is lack of quantitative analysis of allusive resources and mostly focuses on the qualitive analysis, thus increasing the scientificity and accuracy of the qualitative analysis and prompting broad and in-depth conclusions.

#### 5.1.1 Emotion distribution regularity of allusive words

The numbers of positive and negative allusive words were first respectively tabulated as 20,715 and 15,365, respectively. An average difference ratio of 34.82% was found, which is consistent with Hypothesis 2. This can be explained by the fact that in the long-standing and profound Chinese traditional culture, the cultural spirit of being positive and self-improvement has always been advocated. Moreover, throughout Chinese history, the positive energy of striving forward, firmness, and persistence has always been used as the mainstream ideology, so there were more positive than negative allusive words.

**Fig 4:**
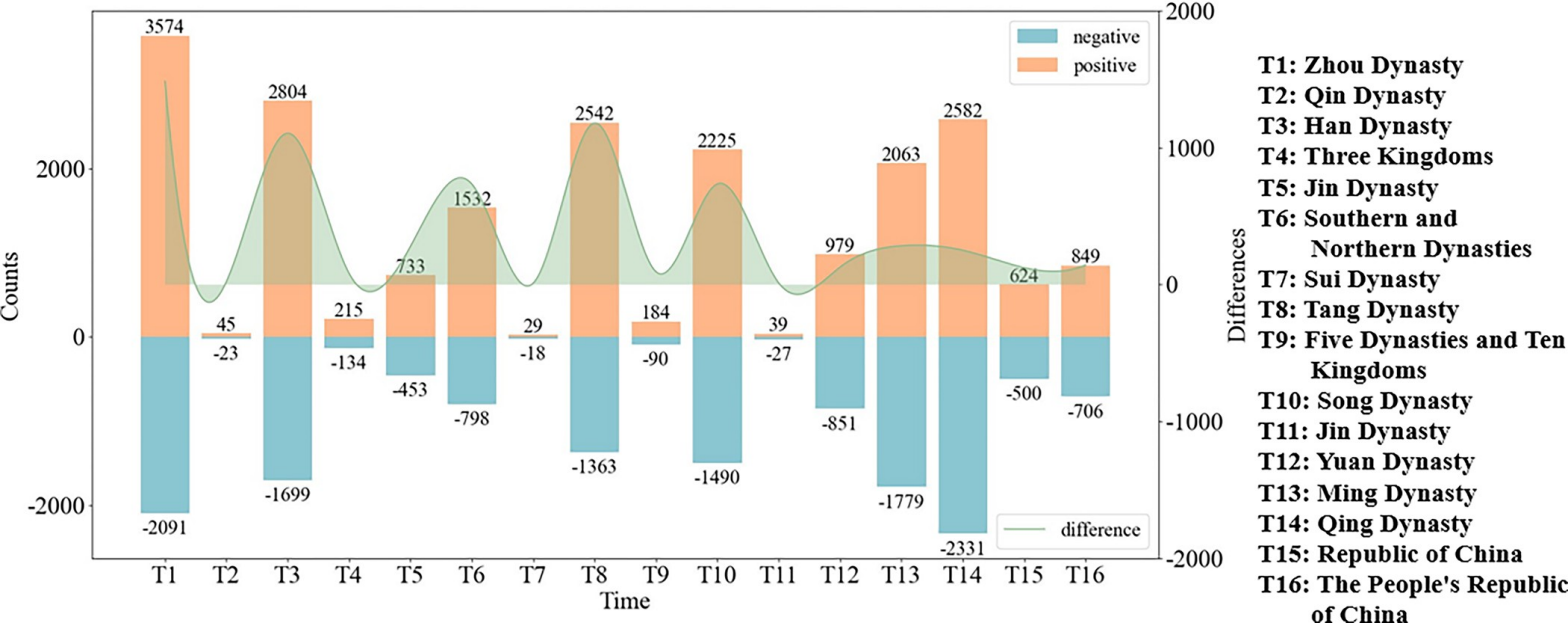
The sentiment distribution based on time transformation.

Next, the emotion distribution of allusive words was explored based on time transformation. Chinese historical stages can be divided into 18 periods, including the Xia Dynasty, the Shang Dynasty, the Zhou Dynasty, etc. Because there are fewer written historical records from the Xia Dynasty and Shang Dynasty, in this study, the Zhou Dynasty was considered the starting point for the analysis of the sentiment distribution of allusive words based on time transformation, and the results are exhibited in Fig 4.

Fig 4 exhibits the numbers of positive and negative allusive words for each historical period via a bar chart, and the difference between the two via a curve diagram. The results reveal the following. First, in each historical period, the number of positive allusive words was found to be greater than the number of negative allusive words, which is consistent with Hypothesis 2. This indicates that in the development of China’s historical process, positive energy has always dominated, and in the face of complex social and natural environments, literary works more often convey moving forward through words, inspire the public to cope positively, convey a positive, healthy, and harmonious artistic atmosphere, and reflect distinctive national characteristics and humanistic spirit.

Also, in terms of quantity, the Zhou, Qing, Han, Tang, Ming, and Song Dynasties had much higher quantities of allusive words than the others. Furthermore, during the Zhou Dynasty, including the Spring and Autumn Period and the Warring States Period, there was no unified feudal regime and no ruling ideology, and scholars of many schools of thought, such as Confucianism, Taoism, and Mohism, were able to write and speak freely. The Han Dynasty’s respect for Confucianism, the establishment of the Imperial College, and Zhang Qian’s mission to the West increased the cultural spread and exchange between the Han Dynasty and the West. Moreover, the unprecedented economic power of the Han Dynasty played a positive role in promoting cultural development. The high economic level, political enlightenment, and the implementation of the imperial examination system during the Tang and Song dynasties laid the foundation for the prosperity of literature, with the most typical literary works being Tang poems and Song lyrics. The literary experience accumulated during the Tang and Song dynasties was inherited by the Ming and Qing dynasties. Furthermore, the prosperity of industrial and commercial towns, as well as the development of bookshops and printing industries, created conditions for literary prosperity, especially for the creation of novels.

From another aspect, there was less literature in the Qin, Sui, and Jin Dynasties. This phenomenon can be attributed to the following reasons. First, these dynasties were shorter. Second, books were burned and Confucian scholars were buried during the Qin Dynasty, leaving fewer historical materials. Moreover, the Jin Dynasty, due to frequent conquests and migrations, also left behind fewer historical records.

#### 5.1.2 Emotion inference

To explore in depth the knowledge embedded in the allusive resources at the fine-grained level, the dichotomous relationships between allusive word-S_*t*_ corpus and allusive word-emotion were utilized to infer the emotion of the S_*t*_ corpus for knowledge inference. According to Hypothesis 3, specific contexts were explored. The S_*t*_ corpora with only one allusive word firstly were removed, and then the remaining S_*t*_ corpora with all allusive words in positive sense, all in negative sense, and a ratio of positive to negative words greater than 1:2 or greater than 2:1 were analyzed. The results are presented in Table 5.

**Table 5:** The text sentiment inference results.

No.	Type	Number	S_*h*_	Number/Ratio of allusive words	Emotion
1	All positive words	1228	*《荀子·富国》* (the Article written by *Xunzi* on his economic ideas)	15	Positive
			*《晋书·杜预传》* (the Biography of *Du Yu* during the Jin Dynasty in China)	9	
			*《汉书·苏武传》* (the Biography of *Su Wu* during the Han Dynasty in China)	8	
			*《尚书·立政》* (the Article on establishing the Official System in China)	3	
			*《登鹳雀楼》* (On the Stork Tower)	2	
2	All negative words	560	*《杀狗劝夫》* (the Script telling the story between human and dogs)	14	Negative
			*《世说新语·轻诋》* (the Article recording the anecdotes and remarks of some famous people in Chinese history)	7	
			*《后汉书·崔寔传》* (the Biography of *Cui Shi* during the Eastern Han Dynasty of China)	4	
			*《北史·崔亮传》* (the Biography of *Cui Liang* during the Northern Wei Dynasty)	3	
			*《大哀篇》* (the Article Slamming the politicians and warlords)	2	
3	P: N = 2: 1	774	*《论语·学而》* (the Article recording Confucius’ conduct oneself in society)	30	Positive
			*《宋书·武帝纪》* (the Biography of the Emperor *Liu Yu* during the Song Dynasty)	11	
			*《南齐书·高逸传》* (the Biography of Gao Yi during the Southern Chi Dynasty)	7	
			*《正气歌》* (the Ode to righteous energy)	4	
			*《新儿女英雄传》* (the Novel telling the story young people guard their homeland)	2	
4	P: N = 1: 2	392	*《鲁斋郎》* (the Script reflecting conflicts between the oppressor and the oppressed in the Yuan Dynasty)	12	Negative
			*《送穷文》* (the Article expressing depressed and frustrated emotions due to lack of achievement)	9	
			*《韩非子·难一》* (the Article written by *Han Feizi* exploring philosophical issues)	5	
			*《陈情表》* (the Memorial to the Emperor stating cases)	3.3	
			*《晋书·段灼传》* (the Biography of *Duan Zhuo* during the Jin Dynasty)	2	

For the type of ‘All positive words’, a search of the data revealed that “*荀子·富国* (the Article written by *Xunzi* on his economic ideas)”is *Xunzi*’s thoughts on the economic aspect of strengthening the capital and enriching the state and his related policies. *Du Yu* was a military man and economist in the Wei and Jin Dynasties who was extremely knowledgeable and accomplished. *Su Wu* was an outstanding diplomat and national hero during the Western Han Dynasty. “*尚书·立政* (the Article on establishing the Official System in China)” is *Zhou Gong*’s admonition to *King Cheng* to be vigilant in peace and to do his best to govern the country. The poem “*登鹳雀楼* (On the Stork Tower)” expresses the poet’s extraordinary ambition to reach for new heights, and reflects the positive and enterprising spirit of the people during the Tang Dynasty. Hence, Hypothesis 3 is valid, and it is plausible to infer the emotion of the S_*t*_ corpus by making use of the relationships between allusive words and the S_*t*_ corpus and emotions for knowledge inference. In addition, it was found that the S_*t*_ corpus for which emotions were obtained via inference was mostly concentrated in the biographies of historical figures, poems, and ancient prose describing the authors’ aspirations or situations. This can be explained by the fact that the content of these texts is thematically distinct and the characters are clear. Thus, the emotions were easier to determine.

### 5.2 Theme association

Based on the dichotomous relationships between allusive words and the E_*t*_ corpus and emotions, the themes of the allusive words in allusive resources were extracted for semantic organization and association.

#### 5.2.1 Allusive word clustering

**Fig 5:**
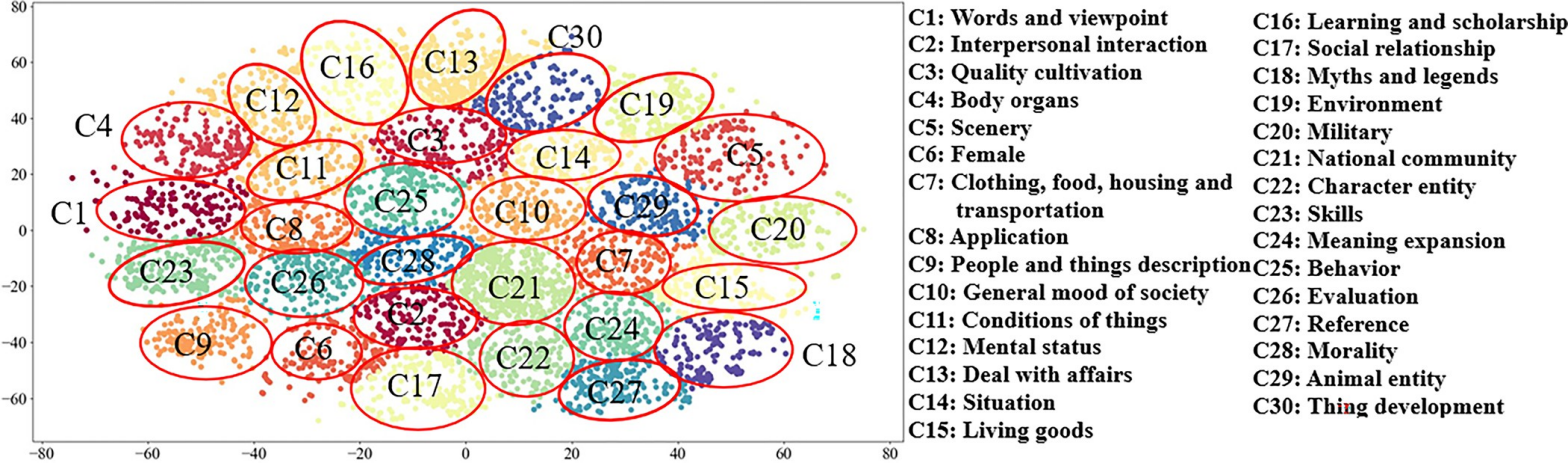
Clusters of allusive words.

First, a pre-trained BERT language model was used to generate the 768-dimension semantic word embeddings of the *E*_*t*_ corpus. Then, to prevent the dimensionality from being too large and spending an excessive amount of time, the t-SNE method was used to reduce the 768-dimension embeddings to 2- dimension, which were then clustered by k-means. When performing clustering, the elbow method was chosen to determine the best *k* value. Then, the value of *k* corresponding to the leveling-off of the decline in SSE was selected as the best *k* value. After calculation, the best cluster number *k* was determined to be 30. To visualize the clustering effect, 200 allusive words in each cluster were randomly selected, and the graph of the clustering effect was plotted. The results are presented in Fig 5.

**Fig 6:**
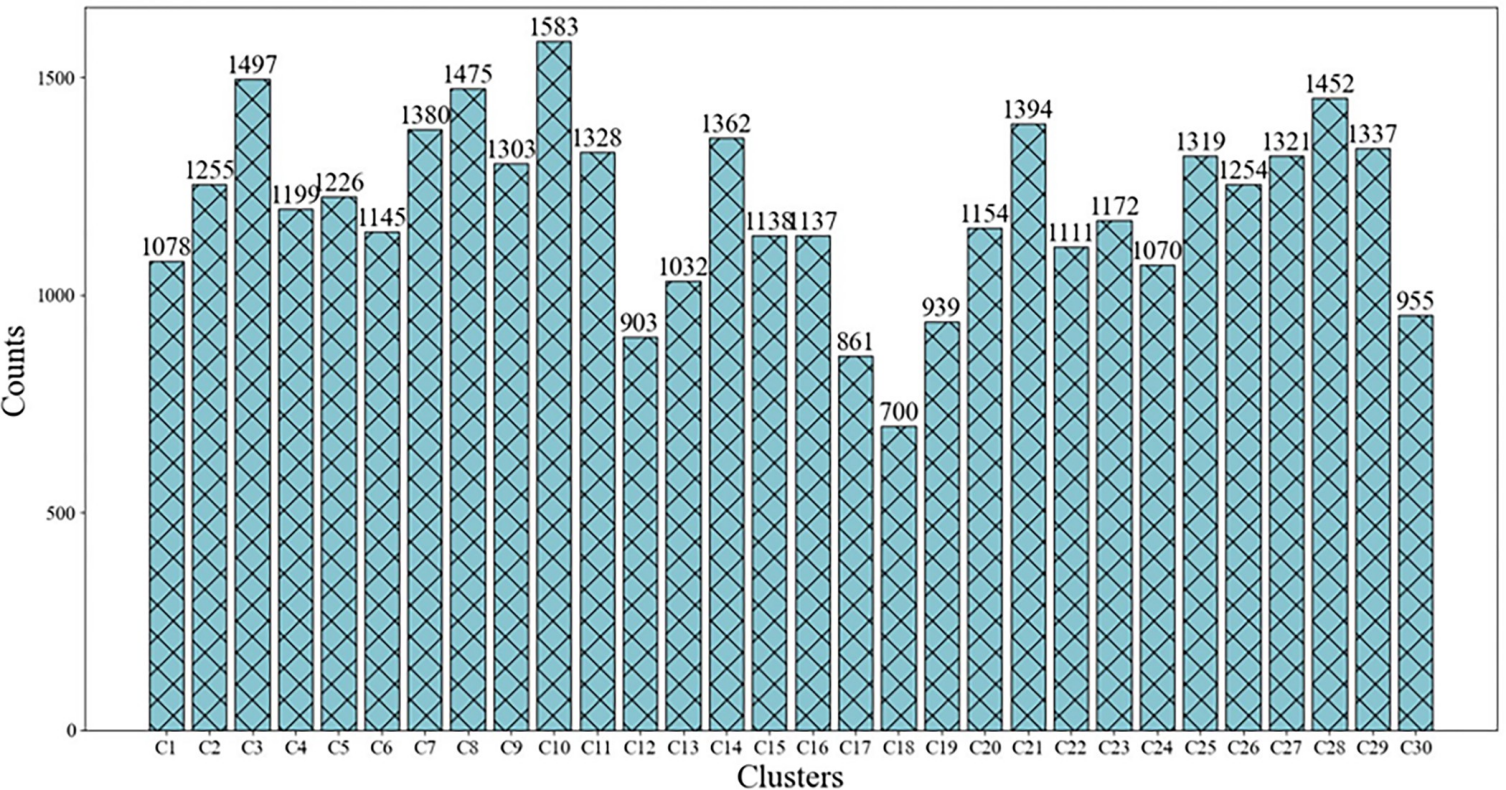
The number of words in each cluster.

As illustrated in Fig 5, better clustering effect was achieved; clustering was well distinguished with clear boundaries between clusters and clusters, and each cluster is tight internally, which has consistency. After the visualization, the numbers of allusive words contained in each cluster were concretely visualized, as displayed in Fig 6.

Fig 6 reveals that the distribution of each cluster was relatively even, and there was no case in which one cluster contained too many allusive words, or in which one class cluster contained too few allusive words. For an in-depth observation, group C4, namely “Body organs,” is taken as an example. The cluster gathered the allusive words including body parts such as the heart, face, eyebrows, etc. However, to the best of the authors’ understanding, these allusive words do not express the same aspects of meaning and reflect different themes. Therefore, the LDA model was employed to extract the topic of each cluster to achieve the organization and association of allusive resources.

#### 5.2.2 Topic extraction

**Fig 7:**
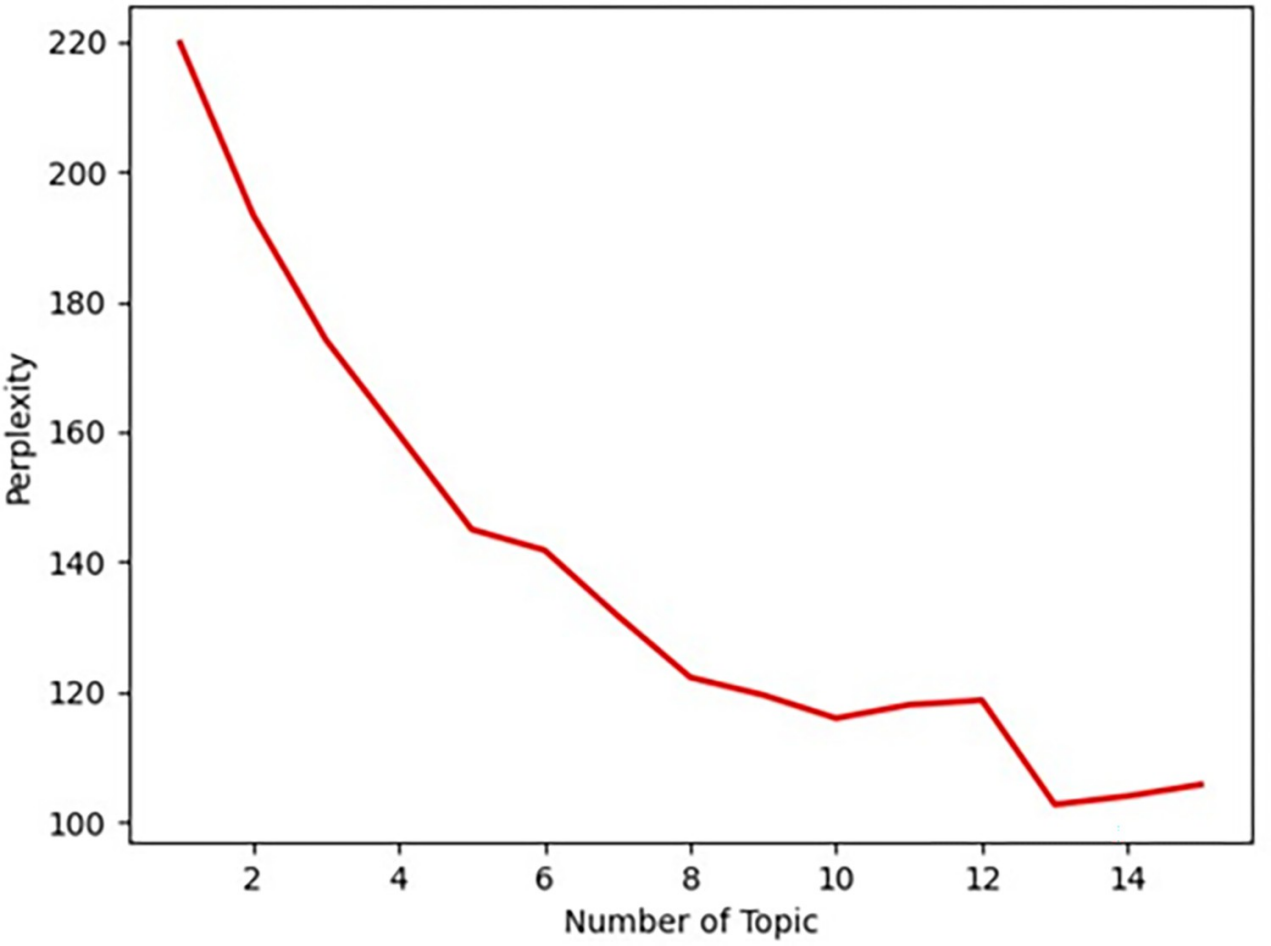
Perplexity.

**Fig 8:**
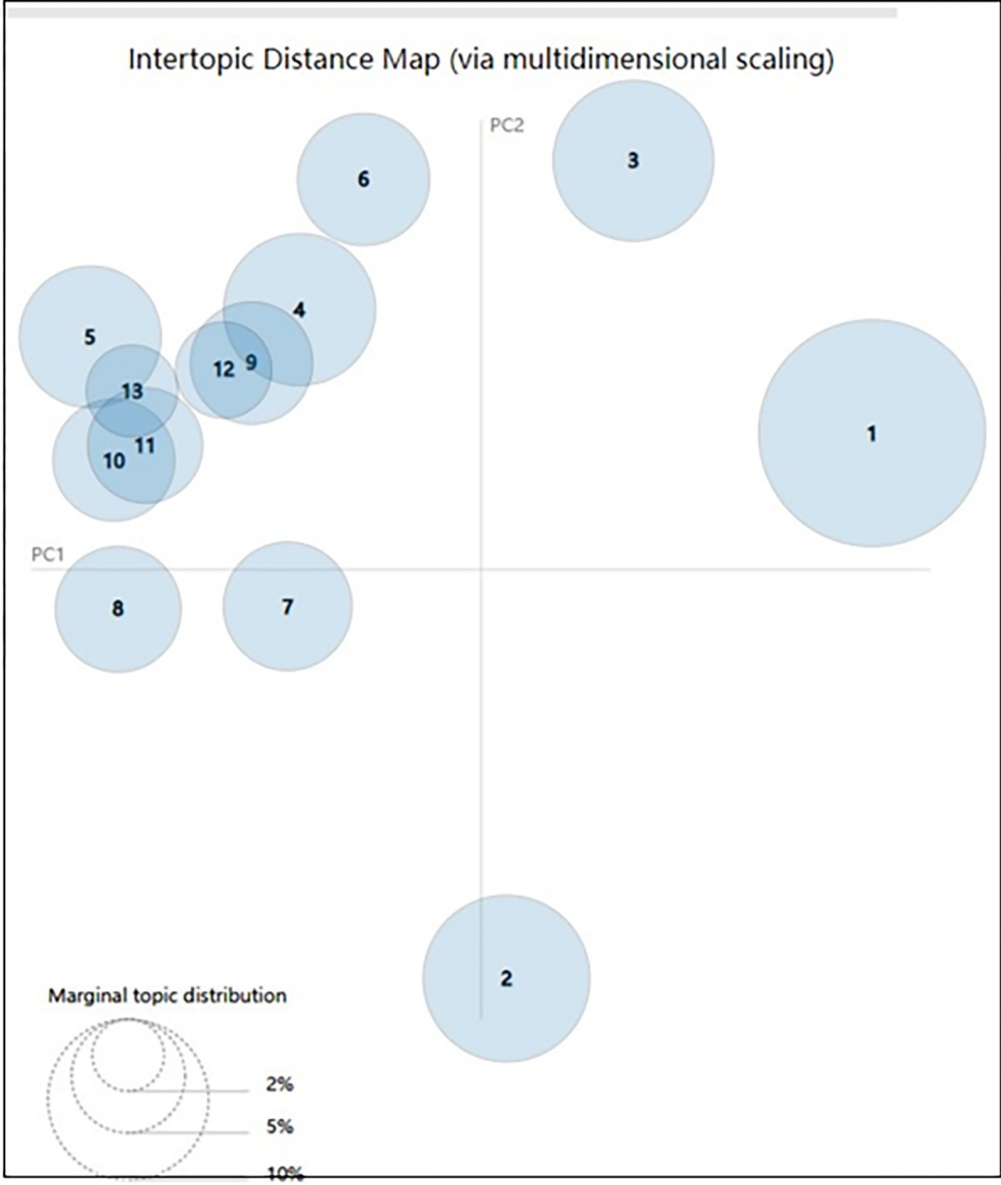
Topic distance.

After generating 30 allusive word cluster groups, the LDA model was utilized for topic extraction. The alpha value was set to 50/k and the beta value was set to 0.01. As an example, the theme extraction experiment was completed with group C4, namely “Body organs.” The allusive word cluster contained 1,199 allusive words after initial clustering. Similarly, the LDA model requires the determination of the optimal number of topics *k*, and the confusion degree was used to determine the optimal *k* value. In this study, the perplexity curve was plotted by taking the *k* value range from 1 to 15, and the results are presented in Fig 7. Moreover, the topic distance graph was plotted, as shown in Fig 8. According to Figs 7 and 8, the best *k* value was determined to be 13.

Then, the 13 topics of group C4 were extracted, the probability of each allusive word belonging to each topic was determined, and the highest probability was chosen as the topic to which the allusive word belonged. The results are reported in Table 6.

**Table 6:** The topic extraction results of group C4.

No.	Cluster	Allusive words (partial)
C4.1	*动作类*(Action)	*张眉努眼* (raise one’s eyebrows and look askance); *缩手缩脚* (bound hand and foot); *横眉怒目* (face others with frowning brows and angry eyes); *畏葸不前* (draw timidly back); *哽咽难鸣* (choke with sobs and be unable to speak)
C4.2	*表情类* (Expression)	*笑容满面* (be all smiles); *破涕成笑* (smile through tears); *怒气冲冲* (a storm of anger); *泪流满面* (be brimming with tears); *呆如木鸡* (dumb as a wooden chicken)
C4.3	*态度类* (Attitude)	*趾高气扬* (be made vain or conceited by success); *颐指风使* (arrogant and bossy); *摇头摆脑* (assume an air of self-conceit); *伏首帖耳* (be subservient to); *昂首扬眉* (raise one’s head proudly)
C4.4	*兴致类* (Interest)	*乘兴而来* (arrive in high spirits); *尽欢而散* (leave only after each has enjoyed himself to the utmost); *鼓舞欢欣* (be elated in spirits); *神色怡然*(look unperturbed); *眉飞色舞* (be overjoyed)
C4.5	*思绪类* (Thoughts)	*眼穿肠断* (look forward with eager expectancy); *目盼心思* (look forward to the mind); *离情别绪* (sad feeling at separation); *抚今痛昔* (evoke memories of the past while living in the present); *鹃啼* (the cuckoo’s cry)
C4.6	*心态类* (Mindset)	*意乱如麻* (be utterly upset); *心急如火* (one’s heart is torn with anxiety); *心向往之* (yearning for somebody or something); *心头撞鹿* (one’s heart goes pit-a-pat); *五内如焚* (one’s heart is rent with grief or torn by anxiety)
C4.7	*气氛类* (Atmosphere)	*喜气冲冲* (full of joy); *天愁地惨* (very miserable); *欢呼雀跃* (shout and jump for joy); *欢呼雷动* (cheers rend the air like thunder); *悲歌击筑* (describe a solemn and bleak atmosphere)
C4.8	*情绪类* (Mood)	*痛心疾首* (resent deeply); *人神共愤* (be hated by everyone); *强颜为笑* (try to show happiness when one is sad); *近乡情怯* (return home with complicated feelings); *感激不尽* (be indebted forever)
C4.9	*情感类* (Affective)	*望洋兴嗟* (bemoan one’s inadequacy in the face of a great task); *令人捧腹* (to make one burst out laughing); *扼腕长叹* (sigh deeply and wring one’s hands); *声泪俱发* (in tearful words); *情意绵绵* (everlasting love)
C4.10	*状态类* (Status)	*痛不欲生* (be overwhelmed with sorrow); *肉跳心惊* (shudder with fear); *满头大汗* (a sweaty face); *头皮发麻* (blood freezes); *如芒在背* (restless and have pins and needles)
C4.11	*心情类* (Feeling)	*惊心怵目* (be shocked at the sight of); *惶恐不安* (be on tenterhooks); *触目兴叹* (be surprised at something); *乐不可言* (extreme pleasure); *乐而忘死* (forget oneself with joy)
C4.12	*形貌类* (Appearance)	*沾沾自喜* (feel complacent over something); *志骄意满* (proud and full of pride); *摇头掉尾* (assume a manner of levity); *吞声忍泪* (swallow the voice and tears); *黯然神伤* (feel dejected to be grieved)
C4.13	*声音类* (Sound)	*啧啧称奇* (click one’s tongue in wonder); *怨声载路* (complaints are heard everywhere); *泣不成声* (sobbing too bitterly to speak); *哽哽咽咽* (groan in sorrow and tears); *人言啧啧* (complaints are whispered in a good-natured way)

After completing the topic extraction, the binary relationships between allusive words and emotions were combined with the theme attributes to build the allusive word-emotion theme relationship database, for which Neo4j was employed for visualization. There are two modes for database display, namely searching for meaning by words and searching for words by meaning.

**Fig 9:**
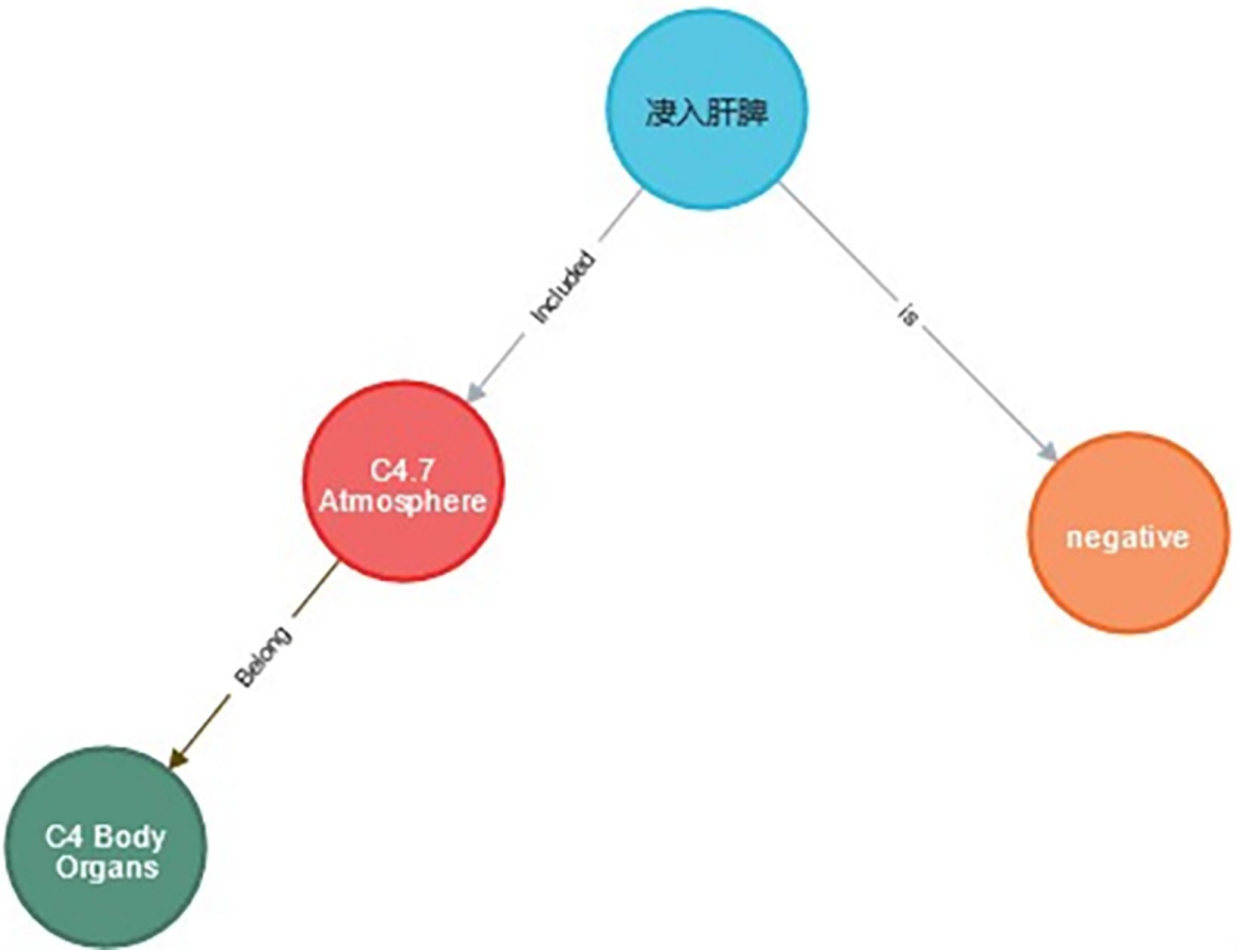
Searching for meaning by words.

“Searching for meaning by words” refers to searching for the theme and emotion of the allusive word in the relationship database. For example, the Cypher query “MATCH p = (:allusive word{name: “*凄入肝脾*”}) RETURN p” was used to search for “*凄入肝脾*” (“be miserable into the liver and spleen”), and the result is shown in Fig 9. It can be seen that the allusive term “*凄入肝脾*” (“be miserable into the liver and spleen”) is negative and belongs to the “atmosphere” theme under group C4, namely “Body organs.”

**Fig 10:**
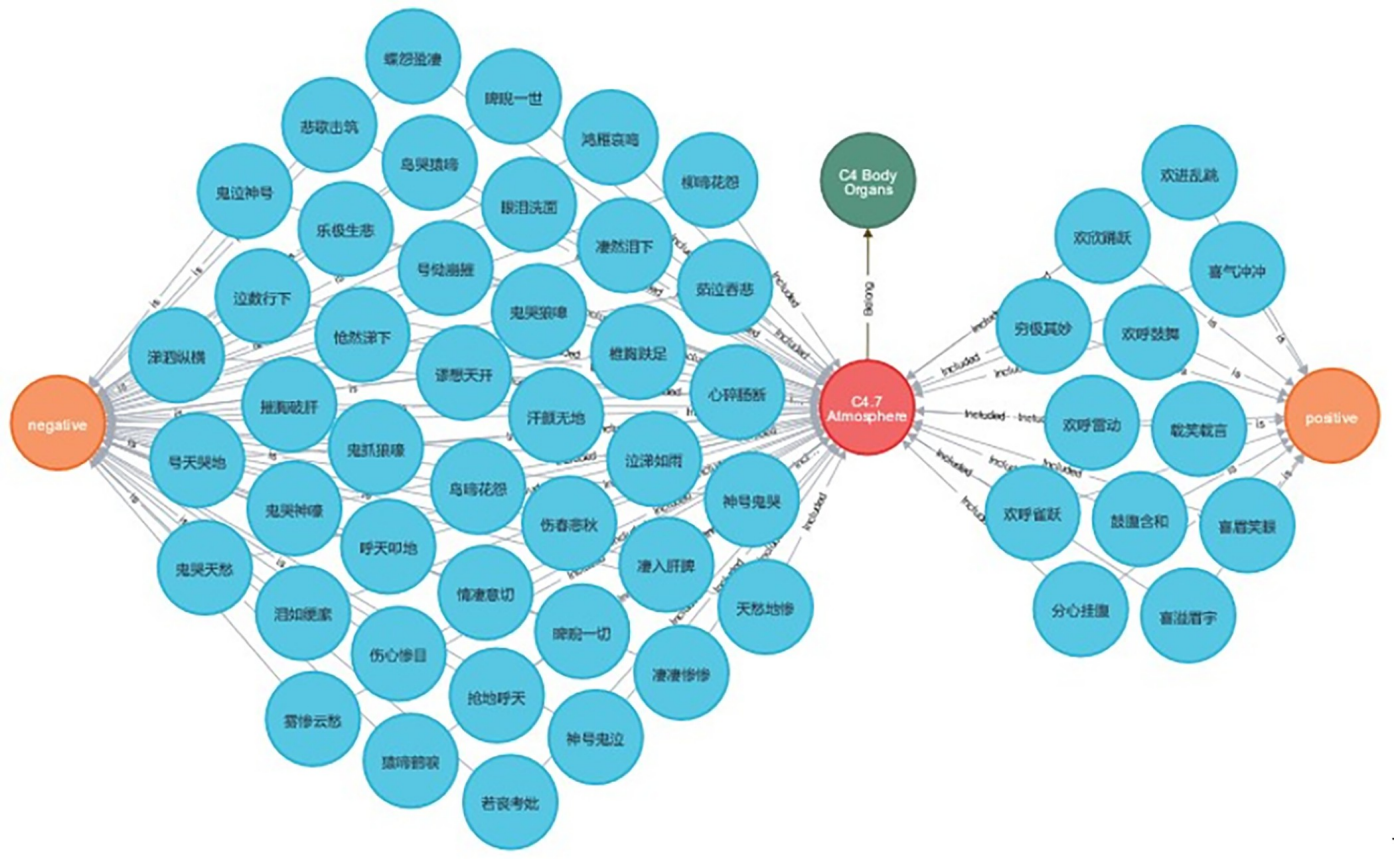
Searching for words by meaning.

“Searching for words by meaning” is a way to find out which positive or negative words are contained in a particular topic in the relationship database. Take topic 7, “atmosphere,” as an example. The Cypher query “MATCH p = (:topic{name: “C4.7 Atmosphere”}) RETURN p” was used to determine which negative and positive words are attributed to topic 7, and the result is shown in Fig 10.

From Fig 10, it can be seen that allusive words such as “*椎胸跌足*” (beat the chest and drop the feet in despair), “*摧胸破肝*” (break one’s chest and liver with great sorrow), “*心碎肠断*” (heart broken), and “*伤心惨目*” (too ghastly to look at) set the mood of desolation and sadness and express negative connotations. In contrast, allusive words including “*喜眉笑眼”* (beaming with joy, very happy), “*喜溢眉宇*” (one’s delight appears at the end of the eyebrow), “*欢呼鼓舞*” (cheers and encouragement), etc., create a pleasant and merry atmosphere and express positive content. Therefore, this confirms the correctness of Hypothesis 4, and proves that fusing the element of the theme can significantly realize the association and organization of allusive resources at the semantic level. Overall, the construction of the allusive word-theme and sentiment relationship database is efficiently able to facilitate users’ acquisition of more knowledge about allusive words, such as emotion polarity and the theme to which the allusive words belong. Moreover, it is capable of offering the knowledge of which positive and negative allusive words belong to a certain topic.

## 6. Discussions

### 6.1 Theoretical implications

This study contributes some theoretical insights. First, it enriches the research related to digital humanities. Digital humanities emphasizes innovative thinking to break through disciplinary boundaries to solve problems, and involves the use of computer technology to help deepen the humanities corpus to discover new problems and expand research horizons. Different from the traditional manual approach utilized by humanities scholars to study massive humanities resources, the automated approach to the semantic association and organization of allusive resources opens up new horizons and provides new ideas for research on allusive resources. Moreover, it activates the multidimensional knowledge embedded in allusive resources and broadens the research boundaries and horizons.

Second, the theory of knowledge organization has been widely applied in libraries, online communities, and scientific and technical literature, but there is a relative lack of research on knowledge organization theory in trope resources. Empowered by deep learning technologies, the research paradigm of knowledge organization is changing. In this study, the degree of organization and the research depth of allusive resources were deepened from the two dimensions of the sentiment recognition of allusive words and the semantic association of emotion topics. This promotes knowledge organization theory and expands the application scope of the theory of the graphical situation archiving discipline, and expands the theoretical influence of the discipline.

### 6.2 Practical implications

This study also has several practical implications. In previous work (Liang, 2015; Huang & Zheng, 2018; Wei et al., 2020), poetry was taken as the research object, and poetry classification, content analysis, and emotion recognition were conducted with the help of allusive words in poetry. In contrast, the present study took allusive words as an important research object and leveraged deep learning algorithms to achieve the automatic emotion recognition of allusive words and construct an allusive word-theme emotion relationship database, which provides the assistance of the organization and semantic association of allusive resources. Furthermore, to promote objective regularity discovery related to allusive resources, the sentiment distribution regularity of allusive words was explored via quantitative analysis methods, and the feasibility of the emotional inference of allusive source texts in specific contexts was investigated. This reduces the subjectivity of previous knowledge related to allusive resources derived from qualitative analysis methods. In addition, the thematic elements of allusive words were integrated, and an allusive word-theme and sentiment knowledge database was designed for the fine-grained and semantic organization of allusive words. This innovates the development and utilization mode of allusive resources, promotes the deep-level development and utilization of allusive resources, and to a certain extent, broadens the application context and practice scenarios of digital humanities research.

## 7 Conclusions and future work

### 7.1 Conclusions

To enable the efficient organization of allusive resources, this study proposed a model of allusive word emotion recognition and application based on text semantic enhancement. For the purpose of text semantic enhancement, the explanatory text was first introduced on the basis of the source text, and the performances of different algorithms were then compared to determine the optimal E-R model for emotion recognition and prediction. Thereafter, the binary relationships between allusive words and the source text, explanatory text, and sentiments were leveraged for regularity discovery and theme association. Regularity discovery contains the sentiment distributions in overall and over time, as well as the text emotion inference. The theme association includes similarity calculation, clustering, topic extraction, and semantic relationship database construction and visualization. The empirical results illustrate that the model proposed in this study is feasible and useful for the organization of allusive resources. To a certain extent, it provides a new way of thinking for the semantic mining of allusive resources.

### 7.2 Future work

In future research, the genre of the source text will be taken into consideration to explore the influence of genre on sentiment recognition, and the sentiment granularity will be refined to further explore the findings related to fine-grained sentiment labels. Moreover, the case that some allusive words can express different emotions in different contexts will be explored to improve our research.

## Supporting information

S1 File All relevant data and codes of this study are openly available in Github at https://github.com/lixm328. (DOCX)
