# Supplementary material for: Research on the sentiment recognition and application of allusive words based on text semantic enhancement
Source: PLoS One. 2024 Nov 4;19(11):e0308944. doi: 10.1371/journal.pone.0308944 (PMC11534255; doi:10.1371/journal.pone.0308944)
Supplement: S1 File — (DOCX) [file pone.0308944.s001.docx]

**S1 File**. All relevant data and codes of this study are openly available in Github at https://github.com/lixm328.
